# Contextual flexibility in the vocal repertoire of an Amazon parrot

**DOI:** 10.1186/s12983-016-0169-6

**Published:** 2016-08-26

**Authors:** Adolfo Christian Montes-Medina, Alejandro Salinas-Melgoza, Katherine Renton

**Affiliations:** 1 Posgrado en Ciencias Biológicas, Instituto de Biología, Universidad Nacional Autónoma de México, Ciudad Universitaria, Mexico City, Mexico; 2 Facultad de Biología, Universidad Michoacana de San Nicolás de Hidalgo, Ciudad Universitaria, Morelia, Michoacán Mexico; 3 Estación de Biología Chamela, Instituto de Biología, Universidad Nacional Autónoma de México, Apartado Postal 21, San Patricio-Melaque, Chamela, Jalisco CP 48980 Mexico

**Keywords:** Animal communication, Lilac-crowned Amazon, Psittaciformes, Signal design rules, Tropical dry forest

## Abstract

**Background:**

Understanding the role of avian vocal communication in social organisation requires knowledge of the vocal repertoire used to convey information. Parrots use acoustic signals in a variety of social contexts, but no studies have evaluated cross-functional use of acoustic signals by parrots, or whether these conform to signal design rules for different behavioural contexts. We statistically characterised the vocal repertoire of 61 free-living Lilac-crowned Amazons (*Amazona finschi*) in nine behavioural contexts (nesting, threat, alarm, foraging, perched, take-off, flight, landing, and food soliciting). We aimed to determine whether parrots demonstrated contextual flexibility in their vocal repertoire, and whether these acoustic signals follow design rules that could maximise communication.

**Results:**

The Lilac-crowned Amazon had a diverse vocal repertoire of 101 note-types emitted at least twice, 58 of which were emitted ≥5 times. Threat and nesting contexts had the greatest variety and proportion of exclusive note-types, although the most common note-types were emitted in all behavioural contexts but with differing proportional contribution. Behavioural context significantly explained variation in acoustic features, where threat and nesting contexts had the highest mean frequencies and broad bandwidths, and alarm signals had a high emission rate of 3.6 notes/s. Three Principal Components explained 72.03 % of the variation in temporal and spectral characteristics of notes. Permutated Discriminant Function Analysis using these Principal Components demonstrated that 28 note-types (emitted by >1 individual) could be correctly classified and significantly discriminated from a random model.

**Conclusions:**

Acoustic features of Lilac-crowned Amazon vocalisations in specific behavioural contexts conformed to signal design rules. Lilac-crowned Amazons modified the emission rate and proportional contribution of note-types used in each context, suggesting the use of graded and combinatorial variation to encode information. We propose that evaluation of vocal repertoires based on note-types would reflect the true extent of a species’ vocal flexibility, and the potential for combinatorial structures in parrot acoustic signals.

**Electronic supplementary material:**

The online version of this article (doi:10.1186/s12983-016-0169-6) contains supplementary material, which is available to authorized users.

## Background

Knowledge of the vocal repertoire of avian species and the association with behaviour enables further understanding of the function and complexity of vocal communication [1–3]. However, the majority of studies on avian vocal communication have been conducted on passerines, with few studies on non-passerines [4–6], effectively narrowing our understanding of the array of signal design strategies for communication in the animal kingdom. Psittaciformes (parrots) are an interesting model for evaluating the behavioural context of the vocal repertoire as parrots have complex social systems that require a similarly complex communication system, and use acoustic communication in a variety of contexts [7], as well as being vocal learners able to acquire acoustic signals through social interaction [8]. Furthermore, parrots use their tongue to modulate sound independent of the source, analogous to that of humans, indicating a speech-like system in the emission of parrot vocalisations [9, 10].

Early studies of psittacine vocal repertoires classified vocalisations by onomatopoeic sound and visual representation in spectrograms [11–14]. Later studies used parametric description of spectrogram features to categorise vocalisations but lacked statistical quantification to objectively differentiate acoustic signals [15–19]. Some studies have attempted to statistically differentiate vocalisations by their temporal or acoustic properties. Univariate analyses of vocalisations found that acoustic signals used by the Blue-crowned Conure (*Aratinga acuticaudata*) varied significantly in emission rate of notes per second, particularly for alarm signals [20]. Long-range alarm calls of the Yellow-faced Parrot (*Alipiopsitta xanthops*) also had a significantly higher emission rate that flight calls, and greater amplitude than sentinel calls [21]. However, few studies have conducted comprehensive statistical analysis of a suite of acoustic traits to reliably differentiate vocalisations. Of these, guttural calls differed from other vocalisations of the Blue-fronted Amazon (*Amazona aestiva*) in note duration and bandwidth [22], while five call-types of the North Island Kaka (*Nestor meridionalis septentrionalis*) were differentiated primarily by call length and secondly by maximum frequency [23].

In general, parrots have been found to present short- and long-range vocalisations [19, 21, 24] of notes with 0–6, and up to ten, harmonics [15–17, 20, 24–26]. Studies of vocal communication of Psittaciformes report from five to 15 calls that can be classified in discrete spectrographic or structural categories [12, 13, 17, 19–21, 23–28], where some vocalisations are given in a variety of contexts, but other vocalisations may be specific to a given behavioural context [19–21, 23–25, 27, 29]. In particular, van Horik et al. [23] determined a significant association of 5 calls of the North Island Kaka with three behavioural contexts of paired, perched, and flying, and Zdenek et al. [25] found a significant association of vocal syllables of the Palm Cockatoo (*Probosciger aterrimus*) with five behavioural contexts.

Selection forces may drive the form or characteristics of vocal signals in accordance with signal design rules to attain optimal communication in a given behavioural context [3]. Design rules state that signals used in differing behavioural contexts should present features of range, locatability, duty cycle (duration and repetition rate), sender identification, within-individual variation, and form-content linkage that optimise coding of the information to be conveyed [3]. The importance of vocal communication in psittacine behaviour and social organisation is reflected by the fact that parrots use vocal signals in a variety of behavioural contexts, yet to date no studies have conducted across-function comparisons of parrot acoustic signals in differing social contexts to identify the combination of design features that could optimise communication, and whether these conform to signal design rules.

Parrots use vocalisations of contrasting characteristics, with long-range acoustic signals where energy is concentrated at low frequencies, and short-range signals of high frequencies [22, 24, 27], suggesting that some design rules may be at play. Behavioural studies of the Lilac-crowned Amazon (*Amazona finschi*) indicate that vocal signals are used to coordinate nesting behaviour by the reproductive pair [30]. However, the characteristics of the vocal repertoire, context specificity of vocalisations, and how these conform to signal design rules, are still unknown. Therefore, in the present study we statistically characterised the vocal repertoire of free-living Lilac-crowned Amazons in distinct behavioural contexts. We aimed to determine whether parrots demonstrated contextual flexibility in their vocal repertoire, and whether these acoustic signals follow design rules that could maximise communication. In accordance with signal design rules [3], we hypothesised that alarm vocalisations would be context-specific, having acoustic characteristics of either flee (low, short, single vocalisation) or assembly (loud, broad, repeated) signals. On the other hand, aggressive threat signals should be more complex being either loud or soft, involve counter-calling, and have characteristics to encode information on body size or motivation. Similarly, nesting vocalisations should be directed at a specific receiver or nest-site, have a high duration or repetition rate, with a diverse repertoire, where both the male and female participate. Finally, we expected vocalisations emitted when perched or foraging to be of short-range, with low diversity and repetition rate, so as to maintain contact with conspecifics but avoid detection by potential predators.

## Methods

We recorded vocalisations of free-living Lilac-crowned Amazons in the tropical dry forest of the Chamela-Cuixmala Biosphere Reserve (19^o^22′N 104^o^56′W to 19^o^35′N 105^o^03′W), on the coast of Jalisco, Mexico. The region has a marked seasonality in rainfall and plant phenology, with precipitation concentrated in 5 months (June to October), and a prolonged dry season [31, 32]. The main vegetation types within the reserve are dense deciduous forest on the hills and slopes, and taller semi-deciduous forest in valleys [33]. Deciduous forest has small trees with a canopy height of 8–12 m, where the majority of plants lose leaf-cover for 5–8 months of the year, whereas semi-deciduous forest has larger trees of 15–30 m height, most of which retain their leaves or drop leaves for 1–3 months of the year [33, 34]. The Lilac-crowned Amazon is endemic to the Pacific coast of Mexico, and nests during the dry season from February to May [30]. Research permits for the study were granted by the Secretaria del Medio Ambiente y Recursos Naturales, Mexico.

### Vocal recording

We recorded vocalisations emitted by parrots at nest-sites, and during opportunistic encounters while they were foraging and resting. Recordings of 61 individuals were made during the morning (07:30–11:00 h) and afternoon (17:00–19:00 h) when parrots are most active [35]. All recordings were made at about 30 m from focal individuals. Parrot vocalisations were recorded with a Marantz PMD 660 or Marantz PMD 670 solid state digital recorders, and a directional ME66/k6 microphone (Sennheiser Electronic) on a shock-mount pistol-grip. Recordings were saved on secure digital or compact flash cards as 16-bitwav files, with a sampling rate of 44.1 kHz or 48 kHz. We then resampled the 48 kHz recordings to standardise them to 44.1 kHz in Goldwave 5.57 (GoldWave Inc.). Recordings were viewed via spectrogram in Raven Pro 1.4 (Cornell Laboratory of Ornithology, New York) with a Hann window size of 592 samples, a 3 dB filter bandwidth of 107 Hz, a frequency grid with discrete Fourier transform size of 1024 samples and grid spacing of 43.1 Hz, and a time grid with a hop size of 59 samples and 90 % overlap, averaging 1 spectra.

### Vocal analyses

We reviewed recordings to extract notes, defined as a continuous sound bordered by a silent interval [36]. Each note was saved in a single file, extracting the note from the original recording with 20 ms of silence at the beginning and the end of the note. ACMM then conducted visual comparison of spectrograms for each note to classify notes in different types. Although we carried out a full account of all notes emitted to evaluate the diversity of acoustic signals, we selected only note-types emitted more than once to describe vocalisations. For statistical analysis we used only note-types that were emitted at least five times across all recordings, and randomly selected five high-quality notes, with low background noise and a high signal-to-noise ratio, for each note-type. We measured five spectrographic variables in Raven Pro 1.4: i) note duration (ms); ii) low frequency (Hz); iii) high frequency (Hz), giving the lower and upper frequency bounds; iv) delta frequency or bandwidth (Hz), being the difference between the upper and lower frequency bounds; and v) number of harmonics. In addition, we used Sound Analysis Pro SA.04 [36, 37] to obtain 6 spectral derivatives for each note: i) mean pitch (Hz), or tone, is a measure of the period of oscillation, or number of cycles made by a sound wave in a unit of time; ii) variance of pitch; iii) mean frequency (Hz), estimates the central tendency of the distribution of power across frequencies; iv) goodness of pitch (Hz), is the peak of the power spectrum for harmonic pitch; v) frequency modulation (deg), is the slope angle of frequency contours; and vi) Weiner entropy, gives a measure of order in the waveform of the sound on a logarithmic scale of 0 (disorder) to minus infinity (complete order). We thereby obtained a total of 11 variables for each note.

Vocalisations were associated with nine behavioural contexts [7, 19, 20, 24, 27]: 1) Nesting activity, when the male returned to the nest after foraging, and called the incubating female who vocalised on leaving the nest-cavity to be fed [30]; 2) Threat interactions of agonistic encounters between conspecifics, usually around the nest; 3) Alarm vocalisations emitted in the presence of potential predators; 4) Foraging, emitted by individuals while foraging in trees; 5) Perched, when parrots were perched inactive or at rest in a tree; 6) Take-off, vocalisations emitted seconds before, during and after flight take-off by parrots; 7) Flight, obtained from flying parrots; 8) Landing, vocalisations of parrots on final flight approach to land in a tree; and 9) Food soliciting, begging by nestlings soliciting food from parent birds, and nesting females soliciting food from males.

Given the difficulties of capturing and marking free-ranging parrots, we considered individual identification based on nest-site ownership for recordings obtained at nest sites. Reproductive pairs of Lilac-crowned Amazons are highly synchronous in nesting behaviour [30]. Therefore for many of the behavioural contexts we used vocalisations recorded at different nest-sites where we could be confident of individual identification. For recordings of behavioural contexts away from nest-sites (foraging, flight), we used only recordings obtained on different days and those that were sufficiently separated by distance among sites to potentially represent different individuals, considering the daily foraging distances travelled by Lilac-crowned Amazons [38]. We were able to distinguish between male and female parrots at nests, as only the female incubates [30]. Therefore, we described both male and female nesting vocalisations, but as this was not possible for other behavioural contexts we did not separate nesting vocalisations by gender for statistical analyses of acoustic parameters among contexts. We collected 75 h of non-continuous recordings over all behavioural contexts, obtaining a total of 8622 notes emitted by 61 Lilac-crowned Amazon individuals that could be spectrographically classified in 152 note-types.

### Statistical analyses

A third of note-types were emitted only once, and were not considered in further analyses, leaving a total of 8571 notes that comprised 101 note-types emitted at least twice. For each of the nine behavioural contexts, we determined the emission rate of notes per second, calculated as the total number of notes emitted divided by time from when the first note was emitted to the last note for that context, which was used as a measure of intensity of vocal activity during the recording period for each behavioural context. We also determined the frequency of occurrence of the most common note-types emitted by adult parrots across all recordings in each behavioural context, and applied chi-square contingency table analysis to determine whether note-types were associated with a specific behavioural context. We calculated adjusted standardised residuals for each cell [39] to determine which notes were used more than expected in each context.

For statistical analyses of acoustic parameters, we eliminated 43 note-types that were emitted less than five times, or had poor quality recordings with a lot of background noise or overlapped other notes. This gave a total of 58 note-types of sufficient sound quality and frequency of emission that were used in statistical parameter analyses among behavioural contexts. We used Generalised Linear Mixed Models (GLMM) fit by maximum likelihood, where we considered behavioural context as a fixed effect, and included individual identity as a random effect across contexts. We excluded the food soliciting context from these analyses, as this included vocalisations of nestlings that were not considered in other contexts. We applied GLMM with log-link using a negative binomial error distribution that showed the best fit to the plot of residuals for the vocal parameters of emission rate, duration, low and high frequencies, and bandwidth. To evaluate number of harmonics among contexts, we employed GLMM with log-link and a Poisson error distribution model that best fit the residuals for the data. We obtained significance values by performing likelihood ratio tests comparing the full model including the effect of behavioural context against the reduced model without the effect. We compared parameter estimates with the intercept, set as the context with lowest mean values. We used the Automatic Differentiation Model Builder (glmmADMB) package [40] to run negative binomial GLMMs, and the lme4 package [41] for the Poisson distribution GLMM, both available in R 3.2.3 [42].

To acoustically discriminate among note-types emitted by adults taking into account individual variation in notes, we selected a data set of 28 note-types that were emitted by more than one individual from the 58 note-types emitted at least five times. We first applied principal component analysis (PCA) on the data set of 28 note-types to convert the 11 spectral and time variables for each note to a reduced set of linearly uncorrelated variables. We then used the Principal Components with eigenvalues >1 in a permuted Discriminant Function Analysis (pDFA) with a nested design that deals with potential non-independence of data when several vocalisations from one individual are included in the data set [43]. Acoustic parameters were considered to aid differentiation among note-types when the observed correct classification was significantly higher (with *P* < 0.05) than the expected correct classification for the null hypothesis of no discrimination among note-types. The expected distribution of the correctly classified signals under the null hypothesis that note-types cannot be acoustically discriminated was obtained performing 1000 permutations. The pDFA was performed using a script for R version 3.0.1 [42] written by R. Mundry, based on the function lda of the R package MASS [44]. Datasets for the statistical analyses are provided in an additional Excel file (Additional file 1). Descriptive statistics are presented as mean with standard error, applying a *P* < 0.05 significance level for statistical analysis.

## Results

### Design characteristics of vocalisations by behavioural context

We provide descriptions of acoustic and behavioural characteristics of each context in an additional Word file (Additional file 2). Overall, a total of 73 note-types were exclusive to a particular behavioural context (Table 1), although the majority of these were emitted infrequently, as shown in Additional file 3: Table S1 that gives the percent emission in nine behavioural contexts for the 101 note-types emitted at least twice (Additional file 3). The greatest variety of 64 note-types were emitted during threat contexts (Table 1). Threat interactions also had the highest number of exclusive notes, where 64 % of note-types emitted during threat interactions were exclusive to this context (Table 1). Vocalisations emitted during threat contexts often involved counter-calling between conspecifics, and were sometimes accompanied by visual displays, such as the wing display where parrots raised both wings in an arc above the body [45], or the tail-fan (Additional file 2). Nesting vocalisations were also highly diverse, with the second-highest number of exclusive notes (Table 1), particularly with regard to male vocalisations where 51.9 % of note-types were exclusively used to call the female from the nest. Among these, the exclusive note Z4 was emitted by males on final approach to the nest, and was spectrographically and acoustically similar to the ‘*grr-uíp*’ vocalisation reported for the Blue-fronted Amazon [24], as shown in an additional figure (Additional file 4) and audio file (Additional file 5). By comparison, alarm vocalisations given in the presence of avian predators such as the Crane Hawk (*Geranospiza caerulecens*) and Collared Forest Falcon (*Micrastur semitorquatus*) had the lowest variety of only nine different note-types, none of which were exclusive to alarms (Table 1). These may be similar to assembly signals as on one occasion we observed 6 Lilac-crowned Amazons flying to congregate with another vocalising pair in response to their alarm calls given on approach by a pair of Collared Forest Falcons.

**Table 1:** Frequency of 101 note-types emitted more than once by Lilac-crowned Amazons in nine behavioural contexts

Behavioural context	Parrot individuals	Behavioural encounters	Total mins recorded	Total notes emitted	Number of note-types	Number of exclusive note-types
Alarm	6	4	3.2	577	9	0
Threat	32	15	35.2	1534	64	41
Flight	28	24	6.7	525	15	5
Take-off	14	14	3.4	238	14	1
Landing	18	29	29.2	1196	24	5
Perched	18	14	19.4	298	16	1
Foraging	6	12	35	451	14	2
Soliciting food (Adult, Chicks)	10 (8, 2)	5 (4, 1)	8.3 (4.7, 3.6)	193, NA	11 (10, 1)	3 (2, 1)
Nesting (Male, Female)	36 (21, 15)	160, 39	92.1 (89.3, 2.8)	3565 (3423, 142)	27 (25, 8)	16 (14, 1)

**Fig 1:**
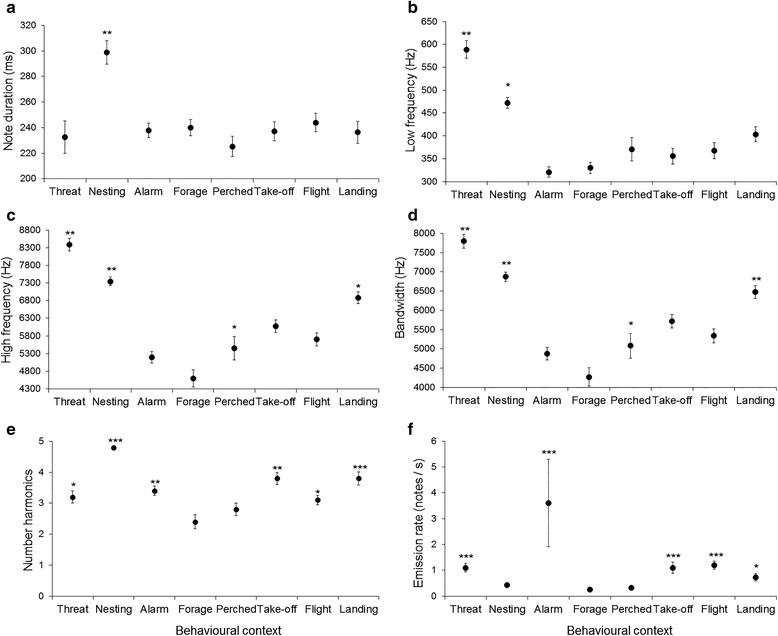
Mean acoustic characteristics of vocalisations emitted by Lilac-crowned Amazons in eight behavioural contexts. (**a**) note duration, (**b**) low frequency, (**c**) high frequency, (**d**) bandwidth, (**e**) number of harmonics, all calculated from 58 note-types emitted ≥5 times across recordings. (**f**) emission rate was calculated considering all notes emitted in each behavioural context. Error bars show standard error. Significant from intercept: * *P* < 0.05, ** *P* < 0.01, *** *P* < 0.001. Forage was set as the baseline intercept in GLMM for all variables except note duration, which was set with the perched context

Acoustic parameter analysis for 58 note-types emitted ≥5 times, determined that including behavioural context as a fixed factor in GLMMs significantly explained variations in note duration (GLMM: χ^2^_7_ = 18.2, *P* = 0.011), low frequency (GLMM: χ^2^_7_ = 74.2, *P* < 0.001), high frequency (GLMM: χ^2^_7_ = 53.5, *P* < 0.001), bandwidth (GLMM: χ^2^_7_ = 46.1, *P* < 0.001), number of harmonics (GLMM: χ^2^_7_ = 50.0, *P* < 0.001), and emission rate (GLMM: χ^2^_7_ = 75.5, *P* < 0.001). The behavioural contexts with most distinct acoustic characteristics were nesting and threat interactions (Fig. 1). Nesting vocalisations had on average notes of longer duration, with the greatest number of harmonics compared to other contexts (Fig. 1). In general, most note-types had three to four harmonics (Mean: 3.4 ± 1.76 harmonics, range = 0–13 harmonics, *n* = 58 note-types), a characteristic of long-range signals in other Amazon parrots [24]. The behavioural contexts of nesting and threat interactions had notes with higher low and high frequencies, and broad bandwidth (Fig. 1). By comparison, alarm vocalisations were distinct in their high emission rate of 3.6 notes/s (Fig. 1), while contexts of perched and foraging had low vocal activity (Fig. 1).

### Classification by note-type

**Fig 2:**
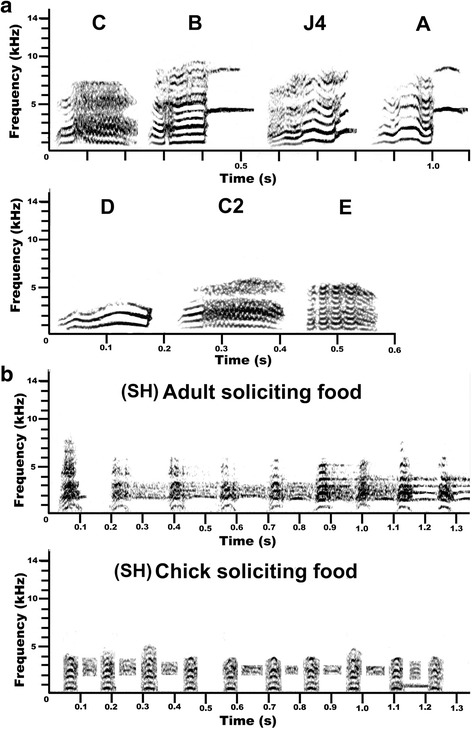
Spectrograms of (**a**) 7 note-types most frequently emitted by Lilac-crowned Amazons, and (**b**) female and nestling begging vocalisations to solicit food

**Fig 3:**
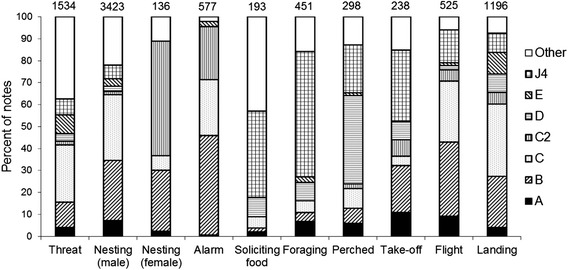
Percent contribution by behavioural context of the 7 note-types most frequently emitted by Lilac-crowned Amazons. Values above columns denote sample size of notes in each context

Considering all notes emitted over all behavioural contexts, seven note-types (Fig. 2a) represented more than 80 % of all notes emitted, and are included in additional audio files (Additional files 6, 7, 8, 9, 10, 11, 12, 13, and 14). Of these, note-types C (26.4 % of notes), B (23.7 %), and J4 (11.5 %) were emitted most frequently, and used in the majority of behavioural contexts. Notes B and C were emitted in all behavioural contexts, but with greater percent contribution in contexts where it was necessary to attract attention of the group or individual such as in alarm, threat, flight, landing, and nesting vocalisations (Fig. 3). By comparison, note J4 was used mainly when foraging, soliciting food, just prior to take-off, and when perched at rest (Fig. 3). The frequency of emission for the seven most-common note-types was significantly associated with behavioural context (χ^2^_54_ = 3984, *P* < 0.001). In particular, note-type J4 was used significantly more when foraging (57 % of notes emitted; cell *z* = 30.3), soliciting food (39 %; cell *z* = 16.7), just prior to take-off (32 %; cell *z* = 9.8), and when perched (22 %; cell *z* = 5.0). The other most common note-type emitted by parrots when perched was note D, which comprised just under half of notes emitted when perched (Fig. 3), and was emitted significantly more than expected in this context (cell *z* = 28.0). Note C was the most common note-type used in threat context (26 %; cell *z* = 5.9; Fig. 3), with the growl-like note E also emitted more frequently than expected in threat interactions (cell *z* = 11.4), but used infrequently in other contexts (Fig. 3). Alarm vocalisations were characterised by note B (45 %; cell *z* = 8.7), although note C2 was also emitted more than expected in alarm contexts (24 %; cell *z* = 20.3), and these two notes comprised almost 70 % of notes emitted in alarm contexts. Note-type C2 was particularly characteristic of female nesting vocalisations (52 %; cell *z* = 26.4), while male nesting vocalisations were characterised by note-types C (30 %; cell *z* = 6.5) and B (27 %; cell *z* = 6.8).

Principal Components Analysis performed with 11 spectral and temporal variables yielded three principal components with eigenvalues >1 for 28 note-types emitted ≥5 times, where each note-type was emitted by >1 individual (Table 2). These components explained 72.03 % of total variance among notes. The variables with greatest loading on Principal Component 1 were variance of pitch, frequency modulation, goodness of pitch, and duration (Table 2). Component 2 was influenced mainly by mean pitch, low frequency, and Weiner entropy (Table 2). The parameters with greatest weight for Principal Component 3 were bandwidth, high frequency, and number of harmonics (Table 2). Discriminant Function Analysis (DFA) for categories of 28 note-types using these three components determined an observed correct classification of 72.7 % that was significantly larger than the expected correct classification for the null hypothesis (Expected = 24.0 %; *P* = 0.001). Similarly, when analysis was controlled by individuals in the pDFA for 28 note-types emitted by more than one individual, the 46.9 % observed correct classification was significantly larger than the 9.6 % expected correct classification by chance (*P* = 0.001).

**Table 2:** Principal Components with eigenvalues >1 for 28 note-types emitted ≥5 times across the study, where each note-type was emitted by >1 individual of the Lilac-crowned Amazon.

	PC1	PC2	PC3
Overall eigenvalues	3.49	2.67	1.76
Explained variation (%)	26.3	23.4	22.3
Variance Pitch	**0.805**	0.384	0.211
Frequency Modulation	**0.794**	−0.325	−0.032
Goodness of Pitch	**−0.704**	0.157	0.293
Duration	**−0.697**	−0.104	0.297
Mean Pitch	−0.165	**0.857**	0.158
Low Frequency	0.083	**0.775**	−0.058
Weiner Entropy	0.531	**−0.659**	−0.158
Mean frequency	0.483	0.585	0.133
Bandwidth	−0.037	0.176	**0.942**
High frequency	−0.026	0.268	**0.916**
Harmonics	−0.287	−0.266	**0.663**

Bold text highlights variables with greatest weighting for each component (*r* >0.60)

## Discussion

### High vocal diversity

The Lilac-crowned Amazon demonstrated a high diversity of 101 note-types emitted more than once, with 58 note-types emitted at least five times, which is one of the largest vocal repertoires so far recorded for Psittaciformes [12, 13, 19–21, 23–29]. However, most studies report calls [12, 13, 15–17, 19–24, 27, 28], or vocalisations [14, 18] that may comprise a number of notes. Fernández-Juricic et al. [24] describe nine call types for the Blue-fronted Amazon, where just the guttural call has at least 23 different note-types [22], and breeding season songs have 17 note-types [24]. Conversational chattering by the Brown-headed Parrot (*Poicephalus cryptoxanthus*) also comprises 23 note-types [17]. Similarly, de Moura et al. [19] classified 9 vocalisations comprised of a total of 36 note-types for the Orange-winged Amazon (*Amazona amazonica*), and May [26] identified 39 acoustic call types for the Grey Parrot (*Psittacus erithacus*). In a similar approach to the present study, Zdenek et al. [25] identified 27 structurally distinct note-types in the vocal repertoire of the Palm Cockatoo. These studies demonstrate that many parrot species have a high diversity of 23–40 note-types commonly used in the vocal repertoire, with the Lilac-crowned Amazon presenting one of the most diverse vocal repertoires, having 58 commonly used note-types.

One explanation for the high diversity of note-types found in our study may be that we have a high recording sample. However, our sample of 75 h of recordings is in the mid-range of that reported by other studies, with lower recording samples of 10 h obtained by Fernández-Juricic and Martella [22] and 30 h by May [26], but larger samples of 100 h reported by Fernández-Juricic et al. [24] and 210 h obtained by Zdenek et al. [25]. Therefore, sample size of recordings is unlikely to explain the high diversity of note-types found in our study. A number of hypotheses may explain this high diversity of vocalisations for the Lilac-crowned Amazon and other parrots, such as acoustic adaptation to forest habitats, social complexity, and ecological characteristics of species.

The Lilac-crowned Amazon may require a larger vocal repertoire to facilitate communication in a complex forest habitat [46, 47]. This could be of particular importance in the tropical dry forest where there is dramatic seasonal variation in phenological characteristics of the forest [32], and the two main habitats of deciduous and semi-deciduous forest have differing vegetation structure [33, 34]. According to the acoustic adaptation hypothesis [46–48], variations in habitat structure affect sound transmission [48, 49] leading to the selection of signals structured to transmit with minimal distortion through native habitat [46–48]. Forests are complex environments, and even slight changes in vegetation structure would have effects on sound transmission with vocal learning species able to rapidly adapt acoustic signals [47], potentially leading to greater diversity in vocal communication [3].

Social organisation may also influence vocal repertoire for species such as parrots, with large, complex social groups. The social complexity hypothesis [50] states that groups with complex social systems require more complex communicative systems to regulate interactions and relations among group members. This variety of social interactions may then lead to the development of large vocal repertoires [50–52]. Parrots exhibit complex social systems [7] where for most species the basic unit is the mated pair, but species such as the Lilac-crowned Amazon also form large communal roosts, smaller foraging flocks, and are territorial around nests in the breeding season [30, 53]. Other parrot species with a large diversity of note-types in vocal repertoires, such as the Brown-headed Parrot, Orange-winged Amazon, Blue-fronted Amazon, and Grey Parrot, exhibit similar flexibility in social organisation [17, 19, 24, 26]. This provides many occasions when individuals may switch group affiliation, requiring mechanisms for recognizing individuals, potentially increasing vocal diversity. Parrots also establish dominance hierarchies by vocal communication [7], requiring complex vocal repertoires to maintain this social complexity. A simple, auditory description of vocalisations by three parrot species in Australia appears to indicate that the species with a more complex hierarchy of groups and individuals has the greatest number of distinct auditory signals [54]. Pidgeon [12] also suggests for five Australian parrot species, that species with more agonistic interactions have a greater number of auditory signals. However, no studies have as yet evaluated the social complexity hypothesis with regard to parrot vocal communication.

Bradbury and Balsby [55] recently suggest that diet-driven social dynamics may explain extensive vocal learning in Psittaciformes. Parrots consume highly variable plant resources of flowers, fruits, or seeds [56], requiring extensive knowledge of potential food resources, and foraging in flexible flocks over a wide area, where vocal learning with the acquisition of new acoustic signals would facilitate identification of individuals with knowledge of food sources [55]. The Lilac-crowned Amazon has a predominantly granivorous diet [57], uses communal roosts [53], and forms foraging flocks with large home-ranges [38]. This species also undertakes seasonal migrations to track food resources [53, 57] that may require a capacity to learn new vocalisations in different regions, habitats, and social groups [58, 59]. Parrot species with vocal learning have been found to modify their vocalisations on relocation to new sites with new social groups [60], and migratory behaviour is associated with larger song repertoires within genera of passerine birds [61]. Therefore, given that parrots maintain vocal learning ability throughout life [7], individuals may encounter and acquire new elements in the vocal repertoire during long-distance movements, and interchange among foraging flocks, leading to high vocal diversity and a low proportion of context-exclusive notes, as found in the vocal repertoire of the Lilac-crowned Amazon.

The Orange-fronted Parakeet (*Eupsittula canicularis*) also inhabits seasonal tropical dry forest, and exhibits fission/fusion flock dynamics [7], but the species has a smaller vocal repertoire [7, 11, 62] compared to the Lilac-crowned Amazon. Therefore, other factors may be influencing vocal diversity of the Lilac-crowned Amazon. One factor may be the larger ranging areas of the Lilac-crowned Amazon with an average home-range estimated to be 4674 ha [38] compared to 666 ha for the Orange-fronted Parakeet which exhibits range lengths of just 6–9 km [63]. Larger movements by the Lilac-crowned Amazon mean that the species is likely to encounter heterogeneous environmental and social conditions that could promote diversity in the vocal repertoire. Another key ecological difference is that the Orange-fronted Parakeet excavates nest-cavities in arboreal termiteria [11] that are generally abundant resources but with only short-term longevity [64, 65]. By comparison, most parrot species, including the Lilac-crowned Amazon, depend on pre-existing naturally-formed tree-cavities [56] that are limited but long-term resources, and exhibit intense intraspecific competition for nest-sites [45]. High vocal diversity may serve to intimidate conspecifics, particularly competitors for nest-cavities, and may reflect selective pressures for a larger vocal repertoire during territorial defense. In support of this, we found greater vocal diversity of Lilac-crowned Amazons in threat interactions with conspecifics around nests compared to other behavioural contexts. Therefore, we consider that competition with conspecifics for scarce, suitable tree-cavity resources may be a contributing factor increasing social complexity and vocal diversity. Hence, the Lilac-crowned Amazon may have a diverse vocal repertoire given that the species inhabits a heterogeneous, seasonal, forest environment, has complex social dynamics including strong intraspecific competition for nest-sites, ranges over a large area, and undertakes long-distance migrations to alternate habitats and regions, all of which may require vocal adaptation to changing environmental conditions and social complexity.

### Design characteristics of the vocal repertoire

Behavioural context significantly explained variations in acoustic characteristics of vocalisations emitted by the Lilac-crowned Amazon. In accordance with signal design rules, threat vocalisations were on average of short duration, with a high emission rate, broad bandwidth, and frequently involved counter-calling, which may encode information on motivation in threat vocalisations. Threat vocalisations were not of low frequencies that could indicate large body size, but had the highest frequency values of all contexts. This may reflect the short-range aspect of threat signals as sender and receiver are generally in close proximity. Furthermore, parrots frequently combined acoustic threat signals with visual displays that may effectively indicate body size, motivation, and an escalation of aggression. Other parrot species have also been reported to use compound signals of high frequency vocalisations with visual displays in threat context [19, 21, 24]. These features correspond to the design rules for threat displays, where a high vocal diversity of notes emitted by Lilac-crowned Amazons, and their combination with visual displays, would permit encoding of additional information on status, body size, intensity, and motivation during threat interactions [3].

Nesting vocalisations were also of high frequency, being short-range signals used between the nesting pair. Nesting vocalisations had the longest note duration which would increase their duty cycle, or percent of time that the signal is active, and broad bandwidth that may have capacity to carry more information in the signal. These are similar to the design rules for courtship signals having both male and female components that are given in a specific sequence [3], although in this case the pair is already mated. This suggests that nesting vocalisations may have a similar role in coordinating activities of the nesting pair; however, experimental evidence is required to determine the function of acoustic signals in nesting contexts.

Alarm signals comprised notes of relatively short duration, with low frequencies, short bandwidth, and had the highest emission rate of 3.6 notes/s. Other parrot species also present alarm vocalisations with high emission rates of short, repeated notes [21, 27, 66]. Wheatcroft [67] determined that various bird species use increased signal repetition rate on approach by a predator, which is recognised as a contextual cue by both adults and nestlings influencing their responses. The low frequencies of alarm signals emitted by the Lilac-crowned Amazon may increase their range through forest habitats as low frequency sounds are less easily absorbed and travel further than high frequency sounds [49]. Features of alarm signals may vary between the extremes of flee and assembly signals, with alert signals having intermediate features [3]. In the case of the Lilac-crowned Amazon, alarm signals given in response to avian predators had features of alert or assembly signals, which are short pulses that are regularly repeated to attract attention and enable location of the sender, rather than flee signals designed to reduce locatability of the sender [3].

Foraging and perched contexts had the lowest emission rates of only 0.3 notes/s that were of low frequencies, short bandwidth, and with few harmonics. This would reduce locatability of individuals where there is a potential cost in attracting predators while individuals are resting, or distracted by foraging. It may also be that vocalisations given in these contexts only need to indicate presence, and therefore do not require greater complexity to communicate more information. It should be noted however, that sample sizes were low for some behavioural contexts, which could be influenced by individual differences, limiting our conclusions on the acoustic characteristics of these contexts. Evidence from playback experiments is also needed to determine the function of acoustic signals used in distinct behavioural contexts.

### Contextual flexibility in use of notes

Note-types could be discriminated by acoustic features, with more than half of all note-types being exclusive to a specific behavioural context, although the seven most common note-types were emitted by Lilac-crowned Amazons in a variety of contexts, but with differing proportional contribution in each context. The common notes B and C were used by Lilac-crowned Amazons with greater frequency in high intensity behaviours of threat, nesting, alarm, and flight, whereas notes J4 and D had greater proportional contribution in low intensity behaviours of foraging, prior to take-off, or when parrots were perched at rest.

Threat contexts had the highest variety of notes, and greatest number of exclusive notes, which may reflect a greater complexity of vocalisations. Zdenek et al. [25] suggest that the complex vocal repertoire of the Palm Cockatoo functions in year-round territorial defense. This has been observed in songbirds, where larger song repertoires are more effective at deterring invaders than small or single-song repertoires [68]. In particular, the growl-like note E that was emitted more frequently than expected in threat context may be similar to the soft vocalisations produced by songbirds in aggressive encounters that are reliable indicators of motivation [69–71].

The next most vocally diverse context for Lilac-crowned Amazons was that of nesting vocalisations, particularly with respect to vocalisations of males calling females out of the nest-cavity. The variety of notes and high proportion of exclusive notes emitted by males when calling the nesting female may permit encoding of individual identity, particularly during incubation of eggs or nestlings when the female lacks visual contact with the male from within the nest-cavity. However, it may be that only a small sample of a vocalisation is required for individual recognition, as Mockingbirds (*Mimus gilvus*) were found to respond to playbacks of conspecifics within seconds, even when presented with only a fraction of the hundreds of song types available per singer [72]. In this sense, the exclusive note Z4 emitted by males on final approach to the nest may contain information on individual identity, alerting the incubating female to her mate’s arrival. The high vocal diversity we found for threat and nesting contexts could be a result of longer recording times, increasing the sample size for these contexts. However, we have equally long recording times for foraging and landing contexts, and these do not show similar vocal diversity, particularly in the number of exclusive notes. Therefore the incorporation of a high number of exclusive notes in threat and nesting contexts may reflect the complexity of interactions and amount of information to be communicated.

By comparison, alarm vocalisations had the lowest variety of note-types, and contrary to expectation, these were not context-specific, but consisted in frequent repetition of three commonly used note-types (B, C, and C2). The Japanese Great Tit (*Parus minor*) has been shown to use acoustically discrete alarm signals for snake predators, but does not use predator-specific alarm signals when mobbing avian predators [73]. Instead of discrete signals for different species of avian predator, birds may vary note repetitions and combinations in compositional syntax to encode information about predator type [73, 74]. It would be interesting therefore to determine whether parrots give different types of alarm signals for terrestrial predators as opposed to avian predators.

Finally, in foraging and perched contexts Lilac-crowned Amazons predominantly used note-types J4 and D, which comprised >60 % of notes emitted and were produced more than expected in these contexts. This contextual flexibility in the use of notes across behavioural contexts may suggest that the vocal repertoire contains a large amount of redundancy in acoustic signals [3]. Alternatively, it may indicate that parrots use graded or combinatorial variation to encode information for different contexts, where the compositional syntax, or the way in which notes are combined, is essential for communicating different messages [74–77].

Our findings on note-type composition in different behaviour contexts suggest that Lilac-crowned Amazons use a variety of strategies for acoustic communication. Other parrot species have also been found to emit calls or notes in a variety of behavioural contexts [13, 20, 22–24], although no studies have determined the cross-functional contribution of commonly used notes in differing behavioural contexts. Functional or contextual flexibility in vocalisations has been determined in non-human primates [78], but there is a paucity of evidence to evaluate the existence of this in other animal groups. Nevertheless, some studies have determined that avian species with small repertoires may use combinatorial structures in compositional syntax to achieve greater communicative complexity [74, 75]. Experimental evidence could determine the acoustic strategies and combinatorial structures employed by parrots for communication in different contexts.

## Conclusions

The Lilac-crowned Amazon presents a diverse vocal repertoire of note-types that are used in a variety of behavioural contexts. This may provide more dimensions for encoding information, which could help the Lilac-crowned Amazon to deal with the constraints imposed on communication within a complex social and natural environment. It is important to evaluate not just the acoustic features and types of notes emitted in each behavioural context, but the compositional syntax of notes used in different contexts [74–77]. Therefore, we propose that evaluation of parrot vocal repertoires based on note-types emitted as the basic unit would reflect the potential vocal diversity of each species. Statistical analysis of the acoustic features of notes, their contribution in each behavioural context, and their combinatorial structures, would reflect the true extent of the species’ vocal flexibility. This would enable comparative studies of vocal diversity among psittacine species to evaluate the relationship of vocal repertoire with habitat structure and social organisation. The cross-functional use of vocalisations by parrots in differing behavioural contexts also makes them ideal species for elucidating signal design rules for differing social functions [3]. Understanding the vocal repertoires of free-living Psittaciformes is essential as a foundation for future research on the extensive vocal learning abilities of parrots [55], the use of combinatorial structures in vocal communication, and the parallels with human language development.

## Additional files

Additional file 1: Datasets for statistical analyses by context and note-type. (XLSX 116 kb) Additional file 2: Descriptions of behavioural and acoustic characteristics in each context. (DOCX 15 kb) Additional file 3: Table S1. Percent emission by Lilac-crowned Amazons in nine behavioural contexts for 101 note-types emitted at least twice across all recordings. (DOCX 24 kb) Additional file 4: Spectrogram of note Z4 emitted by males on approach to the nest. (TIF 593 kb) Additional file 5: 16-bit WAV sound file of note Z4 emitted by a male Lilac-crowned Amazon on final approach within sight of the nest. (WAV 116 kb) Additional file 6: 16-bit WAV sound file of note C emitted by a Lilac-crowned Amazon. (WAV 294 kb) Additional file 7: 16-bit WAV sound file of note B emitted by a Lilac-crowned Amazon. (DOCX 15 kb) Additional file 8: 16-bit WAV sound file of note A emitted by a Lilac-crowned Amazon. (DOCX 24 kb) Additional file 9: 16-bit WAV sound file of note C2 emitted by a Lilac-crowned Amazon. (TIF 593 kb) Additional file 10: 16-bit WAV sound file of note E emitted by a Lilac-crowned Amazon. (WAV 46 kb) Additional file 11: 16-bit WAV sound file of note J4 emitted by a Lilac-crowned Amazon. (WAV 20 kb) Additional file 12: 16-bit WAV sound file of note D emitted by a Lilac-crowned Amazon. (WAV 24 kb) Additional file 13: 16-bit WAV sound file of train of begging notes by a female Lilac-crowned Amazon to the male. (WAV 25 kb) Additional file 14: 16-bit WAV sound file of train of begging notes emitted by nestling Lilac-crowned Amazons in presence of the parent birds. (WAV 19 kb)

## Abbreviations

GLMM: Generalised Linear Mixed Models
PCA: Principal Component Analysis
PC: Principal Component
DFA: Discriminant Function Analysis
pDFA: Permuted Discriminant Function Analysis
