# Supplementary material for: Contextual flexibility in the vocal repertoire of an Amazon parrot
Source: Front Zool. 2016 Aug 26;13(1):40. doi: 10.1186/s12983-016-0169-6 (PMC5000441; doi:10.1186/s12983-016-0169-6)
Supplement: Additional file 2: — Descriptions of behavioural and acoustic characteristics in each context. (DOCX 15 kb) [file 12983_2016_169_MOESM2_ESM.docx]

**Characterisation of vocalisations by behavioural context**

*Nesting*. Vocalisations of males returning to the nest after foraging could be separated in three parts: i) Initial approach of notes emitted by the male while flying towards and within sight of the nest; ii) Calling phase of notes emitted by the male when perched in a tree calling the female from the nest; and iii) notes emitted by the male when accompanying the female from the nest. The initial approach comprised 15 distinct note-types, with at least four introductory notes (D5, M and Z4). In particular, the Z4 note emitted on final approach to the nest was spectrographically and acoustically similar to the ‘*grr-uíp*’ vocalisation reported for the Blue-fronted Amazon [24]. Note Z4 is shown in an additional figure (Additional file 4) and audio file (Additional file 5). Males emitted 19 distinct note-types during the main calling phase, where note-types A, B, C, and C2 (Fig 2a) were most frequently emitted, forming syllables BCC or BC2C2 with a variable number of notes C or C2. Examples of note-types A, B, and C are provided in additional audio files (Additional files 6, 7, and 8). Finally, when the female flew from the nest-cavity, males emitted 5 distinct note-types as they accompanied the female, most frequently note-types B, C, C2, and B4. Overall, the principal note-types emitted by males were C, B, and A, comprising 64% of notes emitted by nesting males.

Female nesting vocalisations were issued to draw attention of the male when she flew from the nest-cavity. The most common note-types used by females were C2 and B (Fig 2a), which comprised 80.1% of all notes emitted by females as they leave the nest. An example of note C2 is included as an audio file (Additional file 9).

*Threat*. These were vocalisations sometimes increasing in intensity to the point of physical contact. The conflict ended with the departure of the intruding pair from the nest area. Vocalisations emitted during threat contexts often involved counter-singing, and may also be accompanied by visual displays, such as the wing display where parrots raised both wings in an arc above the body [45]. In particular, when parrots emitted the three note-types of H6a-H6b-F6, they also exhibited the tail-fan display. Given the high variety of notes emitted during threat interactions, most note-types were emitted infrequently, with note-types B and C (Fig. 2a) occurring most frequently, although these comprised only a third of all notes emitted (37.6%). The next most common note-type in threat contexts was the growl-like note E (Fig. 2a), which comprised 8.6% of notes and is shown in an additional audio file (Additional file 10).

*Alarm*. These were vocalisations emitted by single individuals or pairs in the presence of predators such as the Crane Hawk (*Geranospiza caerulecens*) or Collared Forest Falcon (*Micrastur semitorquatus*). Alarm calls were sometimes followed by agonistic interactions towards the intruder. On one occasion we observed an alarm call by a pair of Lilac-crowned Amazons given in the presence of a pair of Collared Forest Falcons, which caused 6 other Lilac-crowned Amazons to congregate with the vocalising pair. Given the infrequency with which predatory events can be observed in the field, we obtained 3.2 mins recordings of alarm calls. We classified 13 different note-types during alarm calls, although only 9 note-types were emitted more than once (Table 1). The predominant notes emitted during alarm calls were note-types B, C, and C2 (Fig 2a), which together comprised 94.9% of notes emitted during alarm calls.

*Foraging*. We recorded the occasional vocalisations of individuals foraging in trees of *Brosimum alicastrum* and *Astronium graveolens*. These had the lowest emission rate of 0.25 notes/s in 35 min of recordings. We classified a total of 19 note-types emitted while foraging, but only 14 note-types were emitted more than once (Table 1). Of these, the note-type J4 (Fig 2a) represented 57.2% of all notes emitted while foraging. The next most frequent note-types emitted when foraging were notes D and A (Fig 2a), which represented 8.4% and 6.6% of notes emitted respectively. Note-types J4 and D are shown in additional audio files (Additional files 11 and 12).

*Perched*. Parrots emitted occasional short-range vocalisations when perched in trees, comprising a total of 27 distinct note-types, although only 16 note-types were emitted more than once (Table 1). In contrast with other behavioural contexts, the most common note-type was note D, followed by note J4 (Fig 2a), which together comprised 62.1% of all notes emitted when perched.

*Take-off*. Compared with other behavioural contexts, take-off vocalisations had the second-highest emission rate of 1.3 notes/s, with low frequencies and short bandwidth (Fig. 1). Again, three note-types comprised 64.7% of all notes emitted, where J4 was the most common note, followed by notes B and A (Fig 2a). Note J4 was generally emitted while perched, and usually increased in frequency of emission just prior to take-off. Notes B and A were emitted as birds flew from the perch, being functionally similar to the take-off squawk described for other Amazon parrots [14].

*Flight*. We found 15 different note-types emitted in flight, with 5 exclusive note-types (Table 1). Flight calls had in general notes of longer duration, with intermediate frequency, bandwidth, and harmonic values (Fig. 1). Three note-types (B, C, and J4) comprised 76.6% of all notes emitted in flight (Fig 2a).

*Landing*. Vocalisations during landing had a low emission rate of 0.73 note/s, but with a high diversity of 51 distinct note-types, although only 24 note-types were emitted more than once (Table 1). Nevertheless, two note-types comprised over half of notes emitted (note C: 32.8%, note B: 23.3%; Fig 2a), with an additional note E comprising 9.7% of notes emitted (Fig 2a). Among behavioural contexts, landing calls while of short duration, had relatively high frequency, and bandwidth, with a high number of harmonics (Fig. 2).

*Soliciting food*. We obtained 4.7 mins recordings of four females soliciting to be fed by the male. Females emitted two types of begging calls: one where females repeatedly emitted note-type BR; and the second vocalisation type SH where females emitted a rapid sequence of vocalisations with virtually no separation time between notes (Fig 2b), that we were unable to include in the analysis as it was not possible to isolate notes. We also obtained 3.6 mins recordings of three nestlings in one nest soliciting food from the parents. When parents were present, nestlings emitted a similar rapid consecutive SH note-type (Fig 2b), which we also were unable to isolate for parameter analysis. Notes BR and the train of notes termed SH were exclusive to food soliciting and comprised 72.5% of all notes emitted by begging females. The sequence of notes SH (Fig 2b) is spectrographic similar to the ‘*kakaka*’ vocalisations described for the Blue-fronted Amazon [24]. We classified an additional 10 note-types emitted by females when soliciting food. Note J4 was used most frequently by the female to maintain close-range contact with the male prior to being fed. Begging vocalisations by adult females and nestlings are included in additional audio files (Additional files 13 and 14).
