# Supplementary material for: Contextual flexibility in the vocal repertoire of an Amazon parrot
Source: Front Zool. 2016 Aug 26;13(1):40. doi: 10.1186/s12983-016-0169-6 (PMC5000441; doi:10.1186/s12983-016-0169-6)
Supplement: Additional file 3: Table S1. — Percent emission by Lilac-crowned Amazons in nine behavioural contexts for 101 note-types emitted at least twice across all recordings. (DOCX 24 kb) [file 12983_2016_169_MOESM3_ESM.docx]

Table S1. Percent emission in nine behavioral contexts for 101 note-types emitted at least twice by Lilac-crowned Amazons.

| Note type | Alarm | Threat | Flight | Take-off | Landing | Foraging | Soliciting food | Perched | Nesting | | | |
| --- | --- | --- | --- | --- | --- | --- | --- | --- | --- | --- | --- | --- |
|  |  |  |  |  |  |  |  |  | Male | | | Female |
| A | 0.5 | 4.0 | 9.1 | **10.9** | 4.1 | **6.7** | 2.1 | 6.0 | | 7.3 | 2.2 | |
| A5 |  |  |  | 2.1 |  |  |  |  | |  |  | |
| A7 |  | 0.1 |  |  | 0.3 |  | 2.6 |  | |  |  | |
| A9 | 0.2 | 0.2 |  |  |  |  |  |  | |  |  | |
| AA |  | 0.7 |  |  |  |  |  |  | |  |  | |
| AB |  | 0.3 |  |  |  |  |  |  | |  |  | |
| AC |  | 0.9 |  |  |  |  |  |  | |  |  | |
| AD |  | 0.3 |  |  |  |  |  |  | |  |  | |
| AF |  | 0.3 |  |  |  |  |  |  | |  |  | |
| AFa |  | 0.3 |  |  |  |  |  |  | |  |  | |
| AG |  | 0.6 |  |  |  |  |  |  | |  |  | |
| AI |  | 0.3 |  |  |  |  |  |  | |  |  | |
| AL |  | 0.3 |  |  |  |  |  |  | |  |  | |
| AN |  | 0.4 |  |  |  |  |  |  | |  |  | |
| B | **45.4** | **11.6** | **33.9** | **21.4** | **23.3** | 4.2 | 1.6 | 6.7 | | **27.3** | **27.9** | |
| B4 |  | 1.2 | 0.2 | 2.5 |  | 3.6 |  | 0.7 | | 0.2 | 4.4 | |
| B5 |  |  |  | 1.3 | 0.6 | 0.4 |  | 0.3 | |  |  | |
| B6 |  | 0.2 |  |  |  |  |  | 0.7 | |  |  | |
| B8 |  | 0.4 |  |  |  |  |  |  | |  |  | |
| BP |  |  |  |  |  | 1.6 |  |  | |  |  | |
| BQ |  |  |  |  |  | 1.1 |  |  | |  |  | |
| BR |  |  |  |  |  |  | **18.7** |  | |  |  | |
| C | **25.5** | **26.0** | **27.8** | 4.2 | **32.8** | 5.3 | 5.2 | **9.1** | | **29.9** | **6.6** | |
| C2 | **24.1** | 1.8 | 5.1 | 7.6 | 5.4 |  |  | 2.0 | | 1.5 | **52.2** | |
| C6 |  |  |  |  | 0.6 |  |  |  | |  |  | |
| D |  | 3.3 | 1.9 | 8.0 | 8.4 | **8.4** | 8.8 | **40.3** | | 2.4 |  | |
| D5 |  |  |  |  |  |  |  |  | | 0.3 |  | |
| D6 |  |  |  |  | 0.3 |  |  |  | |  |  | |
| E | 2.3 | **8.6** | 1.3 | 0.4 | **9.7** | 2.4 |  | 1.3 | | 3.3 |  | |
| E6 |  | 0.1 |  |  |  |  |  | 0.7 | |  |  | |
| E8 |  | 0.1 |  |  | 0.3 |  |  |  | |  |  | |
| F |  |  |  |  |  |  |  |  | | 6.46 |  | |
| F5 |  | 0.9 |  | 4.6 | 1.4 | 3.8 | 4.7 | 4.7 | | 3.1 | 0.7 | |
| F6 |  | 0.3 |  |  | 0.1 |  |  |  | |  |  | |
| F7 |  | 0.2 |  |  | 0.1 |  |  |  | |  |  | |
| G |  |  |  |  |  |  |  |  | | 1.1 |  | |
| G5 |  |  |  |  |  |  |  |  | | 0.3 |  | |
| G6 |  | 0.4 |  |  | 0.1 |  |  | 0.3 | |  |  | |
| G8 |  | 0.3 |  |  |  |  |  |  | |  |  | |
| H3 |  |  |  |  |  |  |  |  | |  | 2.2 | |
| H5 |  | 0.1 | 0.2 |  |  |  |  |  | |  |  | |
| H5+E5 |  |  |  |  |  |  |  |  | | 3.9 |  | |
| H6ab |  | 0.3 |  |  | 0.1 |  |  |  | |  |  | |
| H8 |  | 0.2 |  |  |  |  |  |  | |  |  | |
| I |  |  |  |  |  |  |  |  | | 0.6 |  | |
| I8 |  | 0.4 |  |  |  |  |  |  | |  |  | |
| J |  |  |  |  |  | 0.7 |  |  | | 2.0 |  | |
| J3 |  |  |  |  |  |  |  | 1.34 | |  | 1.5 | |
| J4 |  | 7.2 | **14.9** | **32.4** | 8.8 | **57.2** | **39.4** | **21.8** | | 6.4 |  | |
| J9 |  | 0.2 |  |  |  |  |  |  | |  |  | |
| K |  | 0.5 |  |  |  | 0.2 |  |  | | 0.7 |  | |
| K6 |  | 0.3 | 0.2 |  | 0.3 |  |  |  | |  |  | |
| K7 |  | 0.2 |  | 0.8 |  |  |  | 0.3 | |  |  | |
| K8 | 0.4 | 1.1 |  |  |  | 2.9 |  |  | |  |  | |
| L |  |  |  |  | 0.1 |  |  |  | | 1.0 | 0.7 | |
| L4 |  | 0.7 | 0.4 |  | 0.4 |  |  | 1.0 | |  |  | |
| L6 |  |  |  |  | 0.3 |  |  |  | |  |  | |
| L7 |  | 0.1 |  |  | 0.1 |  |  |  | |  |  | |
| M |  |  |  |  | 0.3 |  |  |  | | 0.6 |  | |
| M4 |  |  |  |  | 0.1 |  |  | 0.7 | |  |  | |
| N |  | 0.8 |  |  |  |  |  |  | | 0.6 |  | |
| N4 |  | 0.7 | 0.4 | 0.4 | 0.3 |  |  | 1.0 | |  |  | |
| N9 |  | 0.3 |  |  |  | 0.2 |  |  | |  |  | |
| O7 |  |  | 0.6 |  | 0.2 |  |  |  | |  |  | |
| P6 | 0.4 | 1.0 |  |  | 0.1 | 0.7 |  |  | |  |  | |
| P7 |  |  | 0.6 |  | 0.1 |  |  |  | |  |  | |
| P8 |  | 0.2 |  |  |  |  |  |  | |  |  | |
| Q5 |  |  |  |  |  |  |  |  | | 0.1 |  | |
| Q7 |  |  | 0.8 |  | 0.1 |  |  |  | |  |  | |
| Q8 |  | 0.2 |  |  |  |  |  |  | |  |  | |
| Q9 | 0.2 | 3.1 |  |  |  |  |  |  | |  |  | |
| R |  | 0.1 |  |  |  |  |  |  | |  | 1.5 | |
| R7 |  |  | 1.0 |  |  |  |  |  | |  |  | |
| R8 |  | 0.2 |  |  |  |  |  |  | |  |  | |
| R9 |  | 2.4 |  |  |  |  |  |  | |  |  | |
| S8 |  | 0.3 |  |  |  |  |  |  | |  |  | |
| S9 |  | 2.0 |  |  |  | 0.2 |  |  | |  |  | |
| SH |  |  |  |  |  |  | **14.5** |  | |  |  | |
| T6 | 0.2 | 0.1 |  |  |  |  |  | 0.3 | |  |  | |
| T7 | 0.5 |  |  | 0.8 | 0.1 |  | 2.6 |  | |  |  | |
| T8 |  | 0.7 |  |  |  |  |  |  | |  |  | |
| T9 |  | 1.3 |  |  |  |  |  |  | |  |  | |
| U6 |  | 0.1 |  |  | 0.3 |  |  |  | |  |  | |
| U7 |  |  | 0.2 | 1.3 |  |  |  |  | |  |  | |
| U9 |  | 1.1 |  |  |  |  |  |  | |  |  | |
| V3 |  |  |  |  |  |  |  |  | | 0.1 |  | |
| V6 |  | 0.4 |  |  | 0.1 | 0.2 |  |  | |  |  | |
| V7 |  |  | 1.3 |  |  |  |  |  | |  |  | |
| W8 |  | 0.2 |  |  |  | 0.2 |  |  | |  |  | |
| W9 | 0.4 | 3.9 |  |  |  |  |  |  | |  |  | |
| X6 | 0.2 |  |  | 0.8 | 0.2 |  |  | 0.7 | |  |  | |
| X9 |  | 1.2 |  |  |  |  |  |  | |  |  | |
| Y3 |  |  |  |  |  |  |  |  | | 0.2 |  | |
| Y6 |  |  |  |  | 0.3 |  |  |  | |  |  | |
| Y7 |  |  |  |  | 0.3 |  |  |  | |  |  | |
| Y9a |  | 0.3 |  |  |  |  |  |  | |  |  | |
| Y9c |  | 1.0 |  |  |  |  |  |  | |  |  | |
| Z4 |  |  |  |  |  |  |  |  | | 0.9 |  | |
| Z6 |  |  | 0.2 | 0.4 | 0.1 |  |  |  | |  |  | |
| Z8 |  | 0.4 |  |  |  |  |  |  | |  |  | |
| Z9 |  | 3.1 |  |  |  |  |  |  | |  |  | |
