# Supplementary figures and images for: Contextual flexibility in the vocal repertoire of an Amazon parrot
Source: Front Zool. 2016 Aug 26;13(1):40. doi: 10.1186/s12983-016-0169-6 (PMC5000441; doi:10.1186/s12983-016-0169-6)

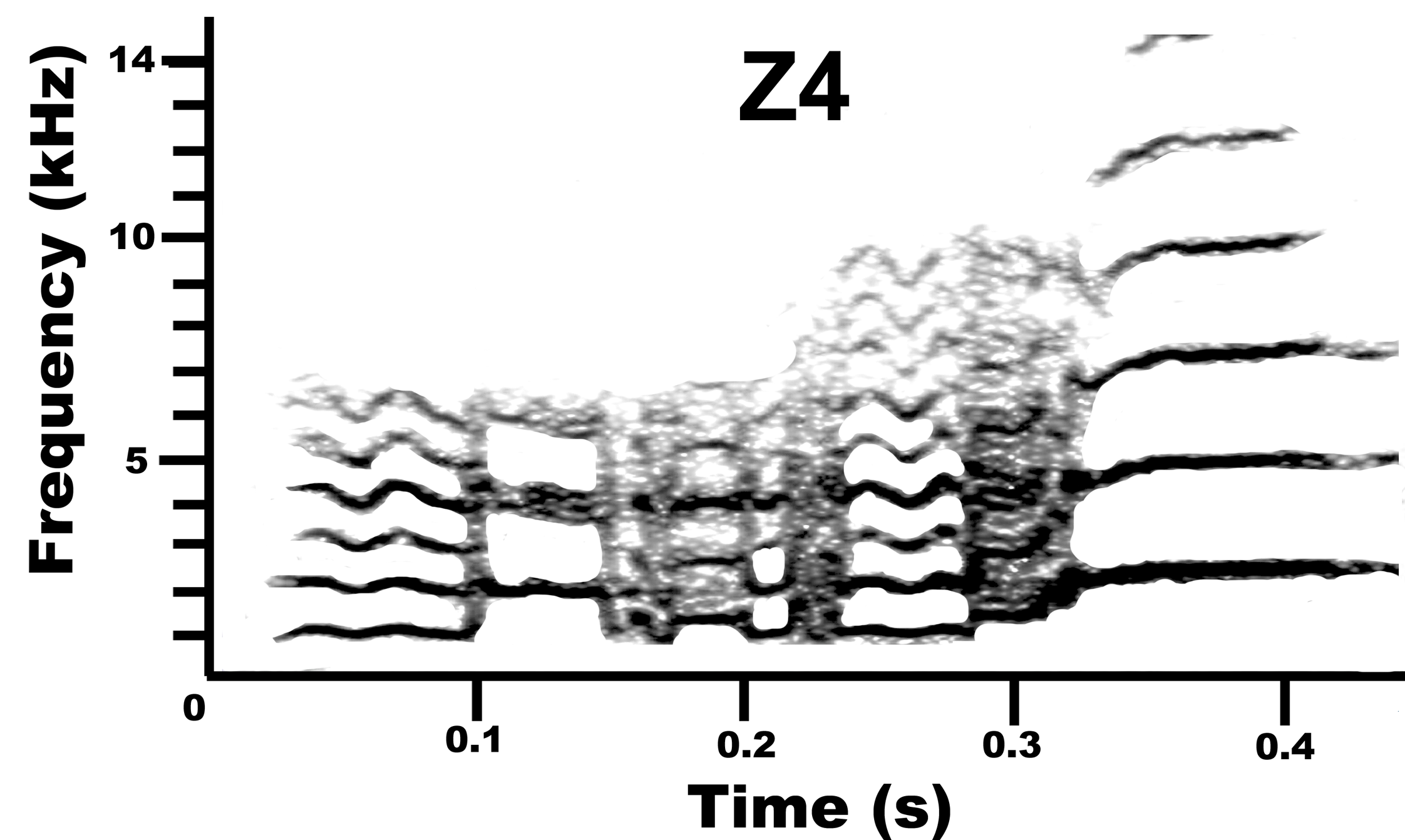

Supplement: Additional file 4: — Spectrogram of note Z4 emitted by males on approach to the nest. (TIF 593 kb) [file 12983_2016_169_MOESM4_ESM.tif]
